# Exploring the links between volcano flank collapse and the magmatic evolution of an ocean island volcano: Fogo, Cape Verde

**DOI:** 10.1038/s41598-021-96897-1

**Published:** 2021-09-01

**Authors:** Mélodie-Neige Cornu, Raphaël Paris, Régis Doucelance, Patrick Bachélery, Chantal Bosq, Delphine Auclair, Mhammed Benbakkar, Abdel-Mouhcine Gannoun, Hervé Guillou

**Affiliations:** 1 Laboratoire Magmas et Volcans, CNRS, IRD, OPGC, Université Clermont Auvergne, 63000 Clermont-Ferrand, France; 2 Laboratoire des Sciences du Climat et de l’Environnement, CEA-CNRS-UVSQ, Université Paris-Saclay, 91191 Gif sur Yvette, France

**Keywords:** Natural hazards, Geochemistry, Volcanology

## Abstract

Mass-wasting of ocean island volcanoes is a well-documented phenomenon. Massive flank collapses may imply tens to hundreds of km^3^ and generate mega-tsunamis. However, the causal links between this large-scale, low-frequency instability, and the time–space evolution of magma storage, crystal fractionation/accumulation, lithospheric assimilation, and partial melting remains unclear. This paper aims at tracking time variations and links between lithospheric, crustal and surface processes before and after a major flank collapse (Monte Amarelo collapse ca. 70 ka) of Fogo volcano, Cape Verde Islands, by analysing the chemical composition (major, trace elements, and Sr–Nd–Pb isotopes) and age-controlled stratigraphy (K–Ar and Ar–Ar dating) of lavas along vertical sections (Bordeira caldera walls). The high-resolution sampling allows detecting original variations of composition at different time-scales: (1) a 60 kyrs-long period of increase of magma differentiation before the collapse; (2) a 10 kyrs-long episode of reorganization of magma storage and evacuation of residual magmas (enriched in incompatible elements) after the collapse; and (3) a delayed impact at the lithospheric scale ~ 50 kyrs after the collapse (increasing EM1-like materiel assimilation).

## Introduction

Among the largest landslides on Earth (tens to hundreds of km^3^) occur on the flanks of ocean island volcanoes such as Hawaii, La Réunion, the Canary or the Cape Verde Islands^1–9^. The influence of external parameters such as climate and sea-level variations has been debated^6,10–12^, but large-scale flank instability of ocean island volcanoes seems closely linked to their volcanic and intrusive history. Indeed, mechanisms of feedback between gravitational instability, structural discontinuities and the intrusive system have been appraised^13–19^. Most of these studies focused on edifice-scale processes, without considering deeper (lithospheric) processes. Numerical simulations^20^ indicate that a massive flank collapse may induce pressure changes at depth down to magma storages in the uppermost mantle (~ 20–40 km), thus accounting for observed variations in magma composition after the collapse^21–26^.

**Figure 1:**
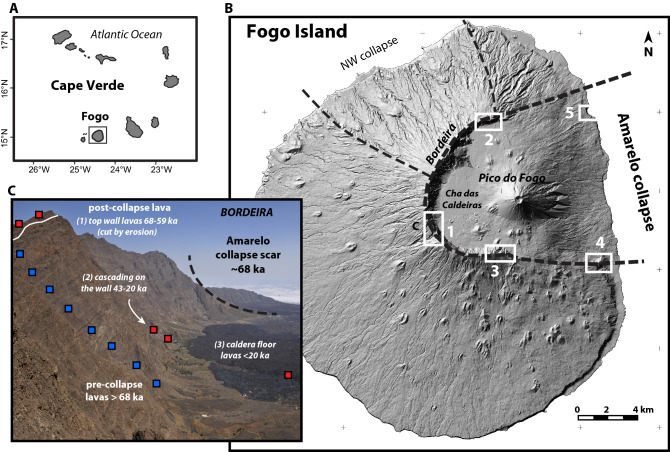
Location map of sampling sites on Fogo Island (Cape Verde archipelago). (1) Western caldera wall; (2) northern caldera wall; (3) southern caldera wall; (4) south-eastern coast; (5) north-eastern coast. Two types of samples are distinguished: samples pre-dating the Monte Amarelo flank collapse (68 ka), and post-collapse samples found on (1) top of the caldera rim, (2) cascading on the wall, or (3) on the caldera floor. Blue and red squares correspond respectively to pre-collapse and post-collapse samples. Shaded relief view derived from the DEMFI (2010) 5 m digital elevation model. Maps realised using QGIS 3.14 (https://www.qgis.org).

This paper aims at tracking potential variations of magma source, partial melting conditions, lithospheric assimilation, levels of storage, crystal accumulation, and fractional crystallisation of lavas emitted at Fogo Island volcano, Cape Verde, before and after a massive flank collapse. The classic top-down approach of the problem (i.e. what are the consequences of the collapse on magma plumbing system and eventually on mantle melting?) is combined with an innovative bottom-up approach (i.e. does the collapse reflect a particular evolution at the source of the magmas, variations of the levels of storage?). We focus on the last 160 kyrs, with a major flank collapse (130–160 km^3^) at ~ 60–70 ka^27–32^. The walls of the horseshoe-shaped collapse caldera (Bordeira on Fig. 1) provides the opportunity for a complete sampling (Table S1) of (a) pre-collapse lavas of the caldera walls, (b) wall-top early post-collapse lavas cut by erosion, (c) post-collapse lavas cascading down wall, and (e) caldera floor lavas (Fig. 1).

## New insights on the volcanic history of Fogo Island

The Cape Verde Islands are considered as the surface expression of a mantle plume at 500–800 km west of the African continental margin^33–35^. The spatial and chronological evolution of volcanic activity, from East to West, is consistent with the slow progression of the African plate over the hotspot since at least the Oligocene, with a decoupling between two distinct branches at ca. 5 Ma^36^. All islands exhibit Quaternary volcanism except Boa Vista and Maio, but Fogo was the only active volcano of the archipelago for the last five centuries^37–39^.

Fogo Island has the morphology of a shield volcano overtopped by a steeper stratovolcano that is truncated by an 8 km-wide horseshoe-shaped caldera opened to the east (Fig. 1: Bordeira). The caldera is bounded on its northern, western and southern sides by steep walls up to 1000 m high offering an open book on the eruptive and intrusive evolution of the volcanic edifice. The origin of the caldera is still debated: a pure gravitational model implying at least one massive flank collapse of the eastern flank of the volcano^27,31,32^, and a hybrid model combining central subsidence (caldera collapse *s.s*.) and flank collapse^30,40,41^. The caldera floor is filled by post-caldera volcanism, including the growth of the Pico do Fogo central cone and historical lavas. The volcanic history of Fogo Island is poorly documented, although different stages of growth separated by major unconformities were distinguished^27,32,38^. Uplifted lavas of the seamount stage are exposed in valleys of the western part of the island together with carbonatite dated 5.1 to 3.2 Ma^42,43^. The early subaerial stage (lower shield), exposed in the northern valleys and in the lower part of the caldera walls, is not chronologically well constrained, with only one lava flow dated 212 ± 20 ka (K–Ar) at the base of the caldera wall^31^. The lavas produced during this stage range from nephelinite and melilitite, to phonolite. Eroded remnants of the lower shield are overlapped by an upper shield, the Monte Amarelo edifice^32^, thus forming a major erosional unconformity that is clearly visible on the walls of the caldera (Fig. S1). The Monte Amarelo stage is characterised by the growth of a ~ 3000 m high central edifice with poorly-defined peripheral rift-zones^32^. The central part of this edifice is densely intruded by dykes and sills, as observed all along the western caldera wall.

Our new K–Ar and ^40^Ar/^39^Ar ages of lava flows (Tables S2 & S3) allow the dating of the early activity of the Amarelo shield at 160 ± 4 ka on the northern caldera wall (Fig. S1), and 135 ± 4 ka on the western wall (Fig. S2). The northern rift-zone was particularly active between 160 and 122 ka. Waning of activity to the north at ~ 120 ka coincides with enhanced activity of the central edifice, including explosive activity. Indeed, thick units of ignimbrites represent half of the material accumulated along the western wall, which contrasts with the absence of explosive products on the northern wall (Fig. S2). Interestingly, it is possible to correlate the ignimbrite units with tephra layers observed in deep-sea cores^44^. A major tephra layer dated 145 ka^44^ might in fact correspond to the ignimbrite observed at the base of the western caldera wall (sample Fo-22 > 135 ka on Fig. S2). Other marine tephra layers at 117 and 88 ka are also concordant with the stratigraphic position of the main ignimbrites found on the western caldera wall. The latest ignimbrite is found on the caldera rim or intercalated with the uppermost pre-collapse lava flows (Figs. S2 & S3). The recurrence of eruptions producing ignimbrites at Fogo, as reported here for the first time, corroborates the existence of caldera collapse structures in the central part of the volcanic edifice^30,40,41^.

## New constraints on the age of the Monte Amarelo collapse

The eastern flank of the Monte Amarelo edifice was destroyed by a massive flank failure leading to the formation of the horseshoe-shaped caldera^5,28,30,32^. Multibeam bathymetry of the eastern flanks of Fogo Island^5,45^ confirmed the existence of a large debris avalanche deposit covering 6820 km^2^ with a volume > 160 km^3^ and a thickness of 100–400 m. The age of the Monte Amarelo collapse was previously inferred from (a) ^3^He exposure ages of pre-collapse (~ 123 ka) and early post-collapse (~ 62 ka) lava flows on Fogo^27^; (b) ^3^He exposure ages of megaclasts transported by the collapse-generated tsunami on Santiago Island (65–84 ka^29^), (c) and K–Ar ages of pre-collapse (~ 60 ka) and post-collapse (43 ka) lava flows of the caldera walls^31^.

Our K–Ar and ^40^Ar/^39^Ar ages give a more precise age of ~ 68 ka for the Monte Amarelo collapse (Fig. 1). Indeed, the uppermost pre-collapse lava flow of the southern caldera wall, which is K–Ar dated 69 ± 2 ka (Fig. S3 & Table S2), is overlain by a post-collapse lava flow which is ^40^Ar/^39^Ar dated 66.9 ± 8.3 ka (Table S3). A post-collapse lava flow overlapping the northern caldera rim has an age of 59 ± 10 ka (Fig. S1), that is concordant with the ages previously obtained on similar wall top lavas^31^. Two post-collapse lava flows cascading from the steep slopes of the caldera wall are dated at 21 ± 2 ka (southern wall), and 20 ± 2 ka (western wall, above a lava previously dated 43 ka^31^). The oldest post-collapse lava flow exposed along the eastern coast is 35 ± 4 ka old (Table S2). Using a first-order reconstruction of the Monte Amarelo edifice before and after its collapse, we estimate a pre-collapse growth rate of 2.2 km^3^/ka (~ 200 km^3^ of lavas produced between 160 and 68 ka), which is relatively high compared to growth rates calculated for the Canary Islands^46–48^. Post-collapse volcanism represents a volume of 74 km^3^ filling the collapse scar at a lower rate of 1.1 km^3^/ka (< 68 ka).

## Sources and processes controlling the composition of the magmas

Silicate lavas of the Cape Verde Islands are ranging from foidites and basanites, to phonolites and trachytes^33,34,49^. Lavas of the southern islands are slightly more alkaline than those of the northern islands^34^. Intrusive and extrusive carbonatites found on São Vicente, Maio, Santiago, Fogo and Brava are interpreted as resulting or deriving from the partial melting of a carbonated oceanic crust^50–53^. Mixing models accounting for the spatial variability of the isotopic signature (Sr–Nd–Pb–He–Os) of the Cape Verde Islands have been proposed^33,34,54–59^. Isotopic and trace element compositions of the lavas of the southern islands suggest a mixture between a moderate HIMU-like (High µ = high ^238^U/^204^Pb) end-member and an EM1-like (Enriched Mantle 1) end-member during magma differentiation at shallow depth^34,56^. The HIMU component is commonly explained by the recycling of a Proterozoic oceanic crust by the mantle plume, while the origin of the enriched component has been debated (lithospheric^56,57^ or asthenospheric^58^). The existence of a third component similar in composition to the Depleted MORB Mantle (DMM) was proposed^57^, but in a very small proportion in the southern islands. Some Cape Verde basalts have also experienced shallow-level contamination by oceanic crustal material (i.e., Jurassic MORB basement)^34^, especially in São Nicolau Island, but this interaction is negligible (possibly non-existent) for Fogo lavas^57^. The overall isotopic (Sr–Nd–Pb) composition of the 48 lava samples collected in Fogo (Fig. S4) is concordant with the model of two main components, with an EM1 component reflecting the assimilation of fragments of continental lithosphere incorporated in the oceanic lithosphere during the opening of the Atlantic Ocean^57^. Interestingly, ^206^Pb/^204^Pb vs*.* ^208^Pb/^204^Pb and ^87^Sr/^86^Sr vs. ^143^Nd/^144^Nd plots of lavas preceding the Monte Amarelo collapse (160–68 ka) do not fully overlap with post-collapse lavas (Fig. S4).

Fogo lavas range from foidites and tephriphonolites (Fig. S5). The ignimbrites observed along the caldera walls have a phonotephritic-to-tephriphonolitic composition, which is concordant with the composition of offshore tephra units^44^. Major-element compositions are controlled by (a) minor variations in the primitive melt composition, and (b) fractionation/accumulation of crystals, mainly pyroxene and olivine. This was previously demonstrated^24,60,61^, and it is also confirmed by the negative correlation between incompatible elements and MgO (Fig. S6), in agreement with highly variable phenocryst content inferred from petrographic observations. Megacrystals are most often not in equilibrium with the melt, and they can be classified as antecrysts from a former magmatic stage. Mass balance calculations (Fig. S7) indicate that the whole rock composition of the lavas implies a crystal fractionation of up to 50% (mostly involving clinopyroxene and amphibole, with lesser proportions of Fe–Ti oxides, apatite, and olivine), and an accumulation of up to 40% (mostly clinopyroxene, olivine ± Fe–Ti oxides). This is in good agreement with the modal compositions of the most commonly observed lavas, where clinopyroxene is often the most abundant megacrysts phase, and with values reported for fractional crystallization of lavas produced by the 1995 eruption^60^. Magma storage and differentiation occurs in the uppermost mantle at depths between 18 and 28 km^60,61^, with short-term secondary storages in the crust^24,60,61^.

## Pre-collapse lavas (160–68 ka)

**Figure 2:**
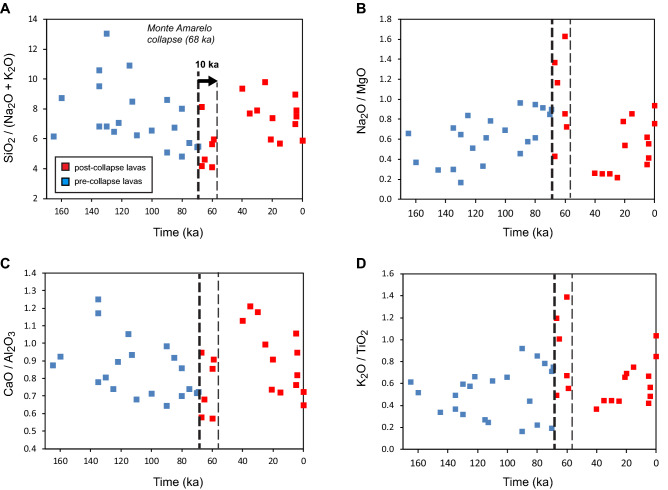
Trends of major element composition of lavas through time, with pre-collapse lavas in blue, and post-collapse lavas in red. Bold dashed line indicates the Monte Amarelo collapse at 68 ka and its inferred short-term impact (thin dashed line at 10 ka after the collapse).

Detailed logging, sampling and dating along the Bordeira caldera walls provide a unique opportunity to address the evolution of magma composition through time, and its possible links with the Monte Amarelo flank collapse (68 ka). Because the major element composition of the primary magmas is almost independent of the source mantle components^24,60,61^, trends of major element composition of the lavas through time indicate a progressive increase of magma differentiation during the 60 kyrs (~ 130–68 ka) preceding the Monte Amarelo collapse (Fig. 2). Lavas become slightly more alkaline with time, as observed on the SiO_2_/(Na_2_O + K_2_O) trend (Fig. 2A). This temporal evolution of magma composition is likely due to increasing pyroxene (± amphibole) fractionation rather than crystal accumulation, as suggested by the Na_2_O/MgO and CaO/Al_2_O_3_ trends (Fig. 2B,C). Variation of K_2_O/TiO_2_ with time displays distinct patterns (Fig. 2D) with high (> 2%) K_2_O lavas on western caldera wall, and low (< 1.5%) K_2_O lavas on the southern flanks. Major element composition somehow varies geographically, with lavas being more differentiated near the paleo-centre of the Monte Amarelo edifice (Fig. S9).

**Figure 3:**
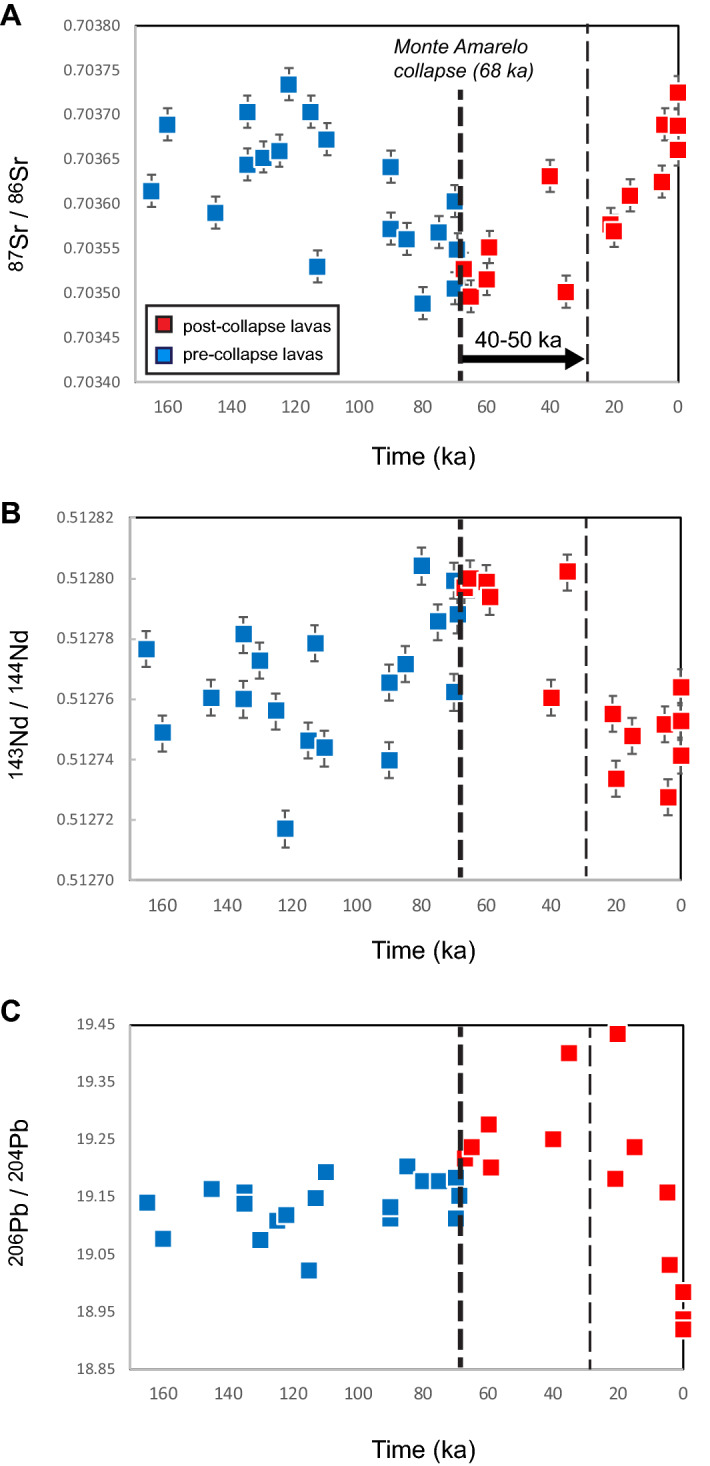
Temporal variations of the ^87^Sr/^86^Sr, ^143^Nd/^144^Nd, and ^206^Pb/^204^Pb isotopic ratios. Bold dashed line indicates the Monte Amarelo collapse at 68 ka and its inferred long-term impact at the lithospheric level (thin dashed line at 40–50 ka after the collapse).

The distinct components in the source of the Fogo magmas can be distinguished based on their Pb–Sr isotope compositions^34,56^. The HIMU component is characterised by a relatively high ^206^Pb/^204^Pb but low ^87^Sr/^86^Sr, and the EM1 component has low ^206^Pb/^204^Pb and high ^87^Sr/^86^Sr. The Pb–Sr–Nd isotopic composition of the lavas displays a significant temporal evolution during the last 160 kyrs (Fig. 3), but unlike the major and trace element trends, these variations are not clearly linked to the Monte Amarelo collapse (Fig. 3). The ^87^Sr/^86^Sr ratio decreases from 160 to 35 ka, whereas the ^143^Nd/^144^Nd ratio shows a slight increase during the same period. The ^206^Pb/^204^Pb is characterised by a progressive increase from 160 to 20 ka, with possibly a more pronounced one after the Monte Amarelo flank collapse (68 ka). Near-collapse lavas are characterised by higher ^143^Nd/^144^Nd and low ^87^Sr/^86^Sr ratios compared to other pre-collapse lavas (Fig. 3). There is thus a long-term increase of the proportion of HIMU component or decline of the EM1 component in the magma source during the pre-collapse period, and this trend persists during 40–50 kyrs after the collapse.

## Post-collapse lavas (< 68 ka)

**Figure 4:**
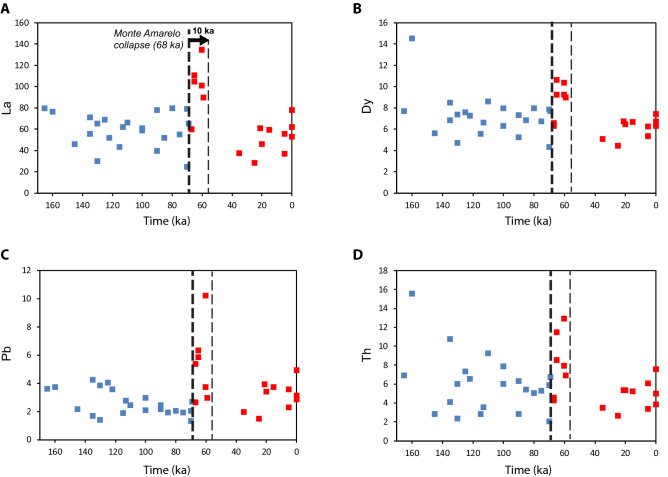
Trends of trace element composition of lavas through time, with pre-collapse lavas in blue, and post-collapse lavas in red. Note the peak of trace elements in early post-collapse lavas (68–59 ka).

Lavas produced soon after the collapse (68–59 ka) share some similarities with the latest pre-collapse lavas in terms of differentiation (Fig. 2A) and isotopic composition (Fig. 3). However, most of these early post-collapse lavas are considerably enriched in incompatible elements (Fig. 4). All these observations converge to a short-term (~ 10 kyrs after the collapse) evacuation of residual magmas characterized by a low degree of partial melting, as observed on a La/Sm vs La plot (Fig. S8). The eruptive vents of these lavas are all located on the rim of the caldera and their products (lava flows) are found in discontinuity on the pre-collapse lavas, although both pre- and post-collapse lavas are cut by retrogressive erosion of the caldera walls.

Late post-collapse lavas (< 59 ka) are characterized by a degree of differentiation that is similar to early (> 100 ka) pre-collapse lavas, i.e. significantly less differentiated than near-collapse lavas (Fig. 2A). This is not directly linked to crystal fractionation, as indicated by similar Na_2_O/MgO for late pre-collapse (< 100 ka) and late post-collapse lavas (Fig. 2B). Peripheral post-collapse lavas filling the collapse embayment on the eastern coast are characterized by olivine rather than pyroxene fractionation (e.g. high MgO and CaO/Al_2_O_3_, but low Na_2_O lavas at 30–40 ka on Fig. 2B,C, see also Fig. S8).

Since 20–30 ka, the ^87^Sr/^86^Sr ratio increased slightly faster than it was decreasing before the collapse (Fig. 3). The ^206^Pb/^204^Pb ratio shows a very fast decline from 19.4 at 20 ka (Table S2: sample Fo-60 dated 20 ka) to < 19.0 for historical lavas, although Mata et al. (2017) reported smaller and shorter-term variations between historical lavas, interpreted as small-scale mantle heterogeneities. After at least 140 kyrs of constant augmentation, the proportion of HIMU component compared to EM1 thus abruptly decreases since 20–30 ka. Although it cannot be definitively proved that the Sr–Nd–Pb isotopic variations illustrated here are connected to the collapse, they most likely correspond to the response to pressure changes at lithospheric depth^20^ favouring EM1-like materiel assimilation that becomes then a dominant process at the magma source.

## Discussion and conclusions

Timescales of astenospheric, lithospheric, and volcanic processes are different, but their respective histories are not disconnected, and the Fogo example provides an original example of interactions between the source and storage of the magmas, and a major volcano flank collapse. In the case of Fogo volcano, major element composition of lavas evidence a progressive magma differentiation due to increasing crystal fractionation during the 60 kyrs preceding the Monte Amarelo flank collapse. This temporal evolution of magma storage in the lithospheric mantle is coeval with the development of shallower reservoirs, including a plutonic complex inside the volcanic edifice itself, and recurrent explosive activity forming ignimbrites. The increasing load of the growing volcano may have promoted magma stagnation, as previously proposed for Teno volcano, Canary Islands^23^. A similar pre-collapse evolution towards more differentiated products was observed on Taburiente volcano, La Palma, Canary Islands^62^, but this trend persisted during the early post-collapse stage (Bejenado lavas) until volcanic activity moved to the South, thus building a new shield (Cumbre Vieja). The central edifice of Tenerife provides another example of such correlation between volcano growth, storage conditions, eruptive processes shifting from effusive to explosive, and flank instability of an ocean island^48,63–65^.

However, the unique dataset presented here for Fogo volcano highlights two different scales of consequences of the flank collapse on lava composition. The delayed response to the unloading effect of the collapse takes ~ 50 kyrs at the lithospheric scale (i.e., the depth at which the EM1-like component is incorporated into the moderate-HIMU-like plume magmas), compared to ~ 10 kyrs at the depth of the main magma storage and crystal fractionation. The shallow and short-term impact (~ 10 kyrs) is evidenced by the composition of early post-collapse lavas (68–59 ka) that are incompatible element-rich. The collapse-induced unloading upset magma storage conditions in the lithospheric mantle (~ 20–30 km) and favoured the release of highly fractionated magma. Major and trace element composition of lavas generated during the last 60 kyrs indicates a degree of differentiation that is similar to that of early (> 100 ka) pre-collapse lavas.

**Figure 5:**
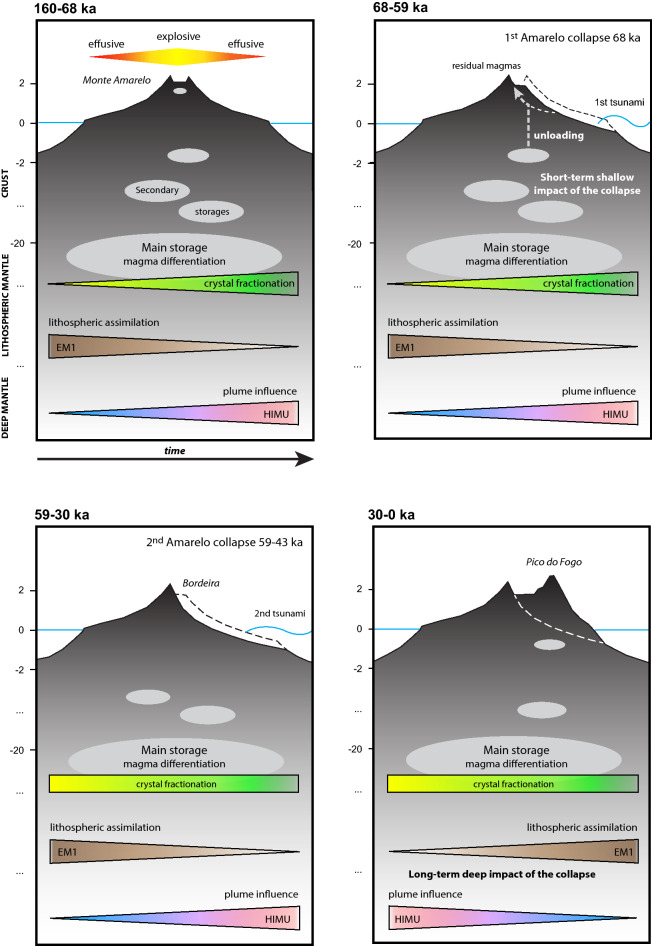
Summary sketch of the magmatic evolution of Fogo island since 160 ka, and possible interactions with Monte Amarelo collapses (68 ka and 59–43 ka).

The temporal and spatial distribution of post-collapse volcanism supports and refines the model of eastward vent shifting^19^, with early post-collapse lavas (68–59 ka) being located along the head of a large failure plane corresponding to the present-day caldera rim (Fig. 5), lavas cascading on the caldera walls between 43 and 20 ka, and later lavas (< 20 ka) on the caldera floor (Fig. 1). This eastward migration may also reflect the retrogressive dynamics of the eastward collapse^28,31,45^. The main collapse is now accurately dated at 68 ka, but secondary collapses may have occurred between 59 and 43 ka. The multistage nature of Fogo flank instability is confirmed by offshore data showing two successive debris avalanche deposits^45^ and it is concordant with what is observed on many other ocean islands^9,65^.

The model proposed here for Fogo should now be compared with other studies using a similar approach, i.e. combining structural, chronological and compositional data^26^. The analytical cost of such studies is high but it is the only way to understand the causal links between volcano growth, instability, and magma storage down to the lithospheric mantle.

## Methods

### Sampling strategy and description of the lavas

A total of 48 lava flows, 11 intrusions (dykes, sills, and intrusive complex of Monte Amarelo), and 2 ignimbrite samples were collected along the caldera walls and coastal cliffs (Table S1, Figs. 1, S1, S2 and S3). The majority of the samples (75%) predate the Monte Amarelo collapse, but almost all the accessible post-collapse lavas were sampled. The geographic distribution of the sampling allows the volcano-stratigraphic units to be dated directly (K–Ar and Ar–Ar ages from this work, and published K–Ar ages^31^) or indirectly (considering their stratigraphic position compared to the dated samples). Six stratigraphic sections were extensively sampled: (1) the northern caldera wall and the Montinho post-collapse lava flow on the caldera rim (Fig. S1); (2) the intrusive complex (dykes and plutonic bodies) of the Monte Amarelo (Fig. S1); (3) the western caldera wall along a 500 m-high via ferrata (Fig. S2); (4) the southeastern caldera wall (Fig. S3); (5) the paleocliff forming the southeastern tip of the collapse scar (Fig. 1); (6) and the cliff of Corvo on the eastern coast (Fig. 1). Four types of lavas were distinguished: aphyric lavas (< 5% of phenocrysts), porphyric lavas (5–40% of phenocrysts, clinopyroxene ± olivine ± amphibole), ankaramites (> 40% of pyroxene and olivine phenocrysts), and intrusive lavas (basanite dykes, nepheline syenites). The mesostatis is micro-crystallised in oxides, pyroxene, olivine, plagioclase and rare felspathoïdes (nepheline, leucite, haüyne). The syenites of the Monte Amarelo intrusive complex are composed of clinopyroxenes, alkali feldspars, nepheline, oxides, and rare biotite. The pyroxene phenocrysts are typically euhedral but often fragmented, and their rims are not in equilibrium with the melt. They display a weak normal zonation (although inverse zonation is also present), with textures of dissolution and recrystallization. Thus, these pyroxenes were most probably captured from a crystal mush during magma storage and ascent.

### Geochronology: unspiked K–Ar technique

Splits of purified groundmass separates from 4 pre-collapse (Fo-36, Fo-43, Fo-31, and Fo-48) and 6 post-collapse samples (Fo-28, Fo-73, Fo-44, Fo-60, Fo-54, and Fo-70) were prepared following the procedure described by^66^. Alteration may cause gain and/or loss of K and Ar and as a consequence may lead to under or overestimated K–Ar ages^67^. To decide if a sample should be used for dating, it is crucial to estimate its degree of alteration. Indeed, after macroscopic and microscopic observations many pre-collapse samples collected along the walls of the caldera were discarded due to their high degree of alteration. The loss-on-ignition values (L.O.I.) range from 0 to 0.4% (Table S1). They are indicative of unaltered samples. The absence of alteration phases in the samples was also verified by observations with a binocular microscope. Isotopic compositions of Ar were determined via the unspiked method^68^ and achieved using the new argon extraction, purification and transfer line recently developed at the LSCE (Laboratoire des Sciences du Climat et de l’Environnement^69^). Groundmass splits (1.0–2.5 g) of samples were wrapped into 99.5% copper foil packets, loaded in the sample holder, which had been turbo-molecular pumped for about 20 h. During the last two hours of that stage, the molybdemium (Mo) crucible was degassed at about 1500 °C until the pressure decreased to 10^–9^ Torr. The sample was then dropped into the Mo crucible and molten at full power of the induction furnace. During the melting stage (i.e. 20 min), the extracted gas was adsorbed by an active charcoal finger at liquid nitrogen temperature. Next to the melting, the gas was released by heating the charcoal at 110 °C and purified via the mutual action of a titanium sublimation pump and a SAES 10 GP-MK3 Zr–Al getter operated at 400 °C. This first step of gas clean-up generally lasted 40 min and was followed by three consecutive exposures of five minutes each of the gas to SAES 10 GP-MK3 Zr–Al getters also operated at 400 °C. The gas was then adsorbed 5 min by a second active charcoal maintained at liquid nitrogen temperature. After adsorption, this sector is isolated. The gas, released at room temperature, was finally cleaned up by a SAES APGP-10 Zr–Al getter operated at 400 °C during another 5 min and introduced into a 180°, 6 cm radius mass spectrometer, equipped with a double Faraday collector. Isotopic analysis was performed on total ^40^Ar contents ranging between 0.5 and 2.5 × 10^–11^ mol. One or multiple manometrically-calibrated doses of atmospheric argon were used to convert beam intensities into atomic abundances and to monitor the atmospheric correction. The manometric calibration is based on periodic, replicate determinations of the international dating standard HD-B1 (24.21 ± 0.32 Ma^70,71^). Uncertainties for the K and Ar data reported in Table S2 are 1σ (analytical only), and consist of propagated and quadratically averaged experimental uncertainties arising from the K, ^40^Ar (total), and ^40^Ar* determinations. All uncertainties on the ages are given at 2σ.

### Geochronology: ^40^Ar/^39^Ar method

In addition to K–Ar dating and to better constrain the age of the collapse, we have achieved a ^40^Ar/^39^Ar age determination on sample Fo-01 (Table S3), previously K–Ar dated at 86 ± 3 ka^28^. Indeed, the K–Ar age of this post-collapse sample was questioned^29^. ^40^Ar/^39^Ar measurements on similar groundmass samples of FO-01 may allow verifying if the K–Ar age was in error by excess. Irradiation, extraction, gas clean-up procedures, mass spectrometric measurements and blank corrections, are detailed in ^66^. The total decay constants given by ^72^ and the ^40^Ar/^36^Ar atmospheric ratio at 298.56^73^ were used for age calculations. The precision of the mass discrimination correction was monitored by daily measurements of air argon and is about 0.15% (2σ standard deviation for multiples of experiments). Neutron fluence (J) was monitored by co-irradiation of Acs crystals^74^ placed in three pits encircling each sample. The J value for each sample was determined from 6 single crystal laser fusion analyses of Acs. Corresponding J values were calculated using an age of 1.1840 ± 0.0007 Ma^75^.

### Major and trace elements chemistry

Major element compositions (Table S1) were analysed by ICP-AES (Inductively Coupled Plasma-Atomic Emission Spectrometry) at LMV (Clermont-Ferrand, France) after alkaline melting with lithium borate and nitric acid dissolution (100 mg of sample)^76^. BHVO-1 standard was also analysed during the analytical session. For trace elements analysis (Table S1), samples were dissolved in a HNO_3_–HF mixture, heated for 24 h and then evaporated. After dissolution, fluoride precipitates were dissolved with several cycles of additions of 7 N HNO_3_ and 6 N HCl and evaporations. Additionally, major element composition of ignimbrite and pumice samples (Table S1) was determined using an electronic microprobe (SX100 CAMECA) at LMV. An accelerating voltage of 15 kV was combined with a beam current of 10 nA for a beam diameter of 1 μm.

Whole-rock trace elements were obtained by solution Inductively-Coupled Plasma Mass-Spectrometry (ICP-MS, Agilent 7500) at LMV. The BHVO-2 standard was measured every five samples, and the reproducibility was determined with BCR-2 and CMS standards^77^.

### Sr–Nd–Pb isotopic composition

One hundred milligrams of bulk-rock powder were leached with hot 6 M-HCl (75 °C) during 3 h following the protocol described in ^57^, acid-digested with HF-HNO_3_, and passed through the chromatography procedure of ^78^. Strontium, Nd and Pb analytical blanks were all negligible with respect to sample contents. Strontium isotopic measurements were performed on a Finnigan Triton thermo-ionization mass spectrometer (TIMS), Pb measurements on a Thermo Fisher Neptune Plus Multiple Collector—Inductively Coupled Plasma—Mass Spectrometer (MC–ICP–MS), and Nd measurements used both instrumentations (Table S1). Strontium isotope ratios were mass-fractionation-corrected to ^86^Sr/^88^Sr = 0.1194 and normalized to ^87^Sr/^86^Sr = 0.71025 for the NIST SRM987 standard. Neodymium isotope ratios were mass-fractionation-corrected to ^146^Nd/^144^Nd = 0.7219 and normalized to ^143^Nd/^144^Nd = 0.512100 for the JnDi-1 standard. Repeated TIMS analyses of the two standards during the course of the study gave ^87^Sr/^86^Sr = 0.710242 ± 18 (2sd, n = 33) and ^143^Nd/^144^Nd = 0.512104 ± 6 (2sd, n = 17). Lead mass discrimination was corrected by standard bracketing of samples with NBS-981 standard using values of ^79^ recalculated to ^208^Pb/^206^Pb = 2.1677^80^. Average NBS-981 deviations from drifts observed during the different day-sessions of analysis were in the ranges 35–120, 45–190 and 65–255 ppm for ^206^Pb/^204^Pb, ^206^Pb/^204^Pb and ^206^Pb/^204^Pb ratios. This corresponds to maximum errors of ± 0.002, 0.003 and 0.010, respectively.

## Supplementary Information

Supplementary Figure S1.

Supplementary Figure S2.

Supplementary Figure S3.

Supplementary Figure S4.

Supplementary Figure S5.

Supplementary Figure S6.

Supplementary Figure S7.

Supplementary Figure S8.

Supplementary Figure S9.

Supplementary Information.

Supplementary Table S1.

Supplementary Table S2.

Supplementary Table S3.

## Data Availability

All data generated or analyzed during this study are included in this published article (and its Supplementary Information files).
