# Supplementary figures and images for: Exploring the links between volcano flank collapse and the magmatic evolution of an ocean island volcano: Fogo, Cape Verde
Source: Sci Rep. 2021 Sep 1;11:17478. doi: 10.1038/s41598-021-96897-1 (PMC8410878; doi:10.1038/s41598-021-96897-1)

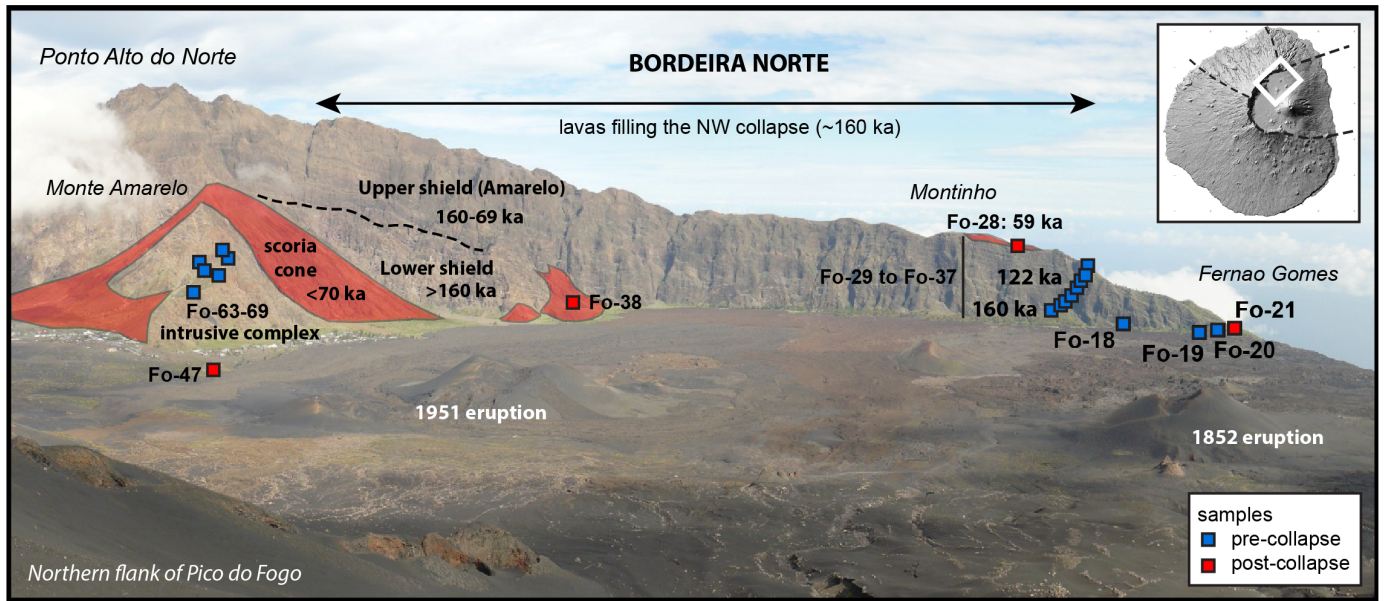

Supplement: Supplementary file 1 — Supplementary Figure S1. [file 41598_2021_96897_MOESM1_ESM.pdf]

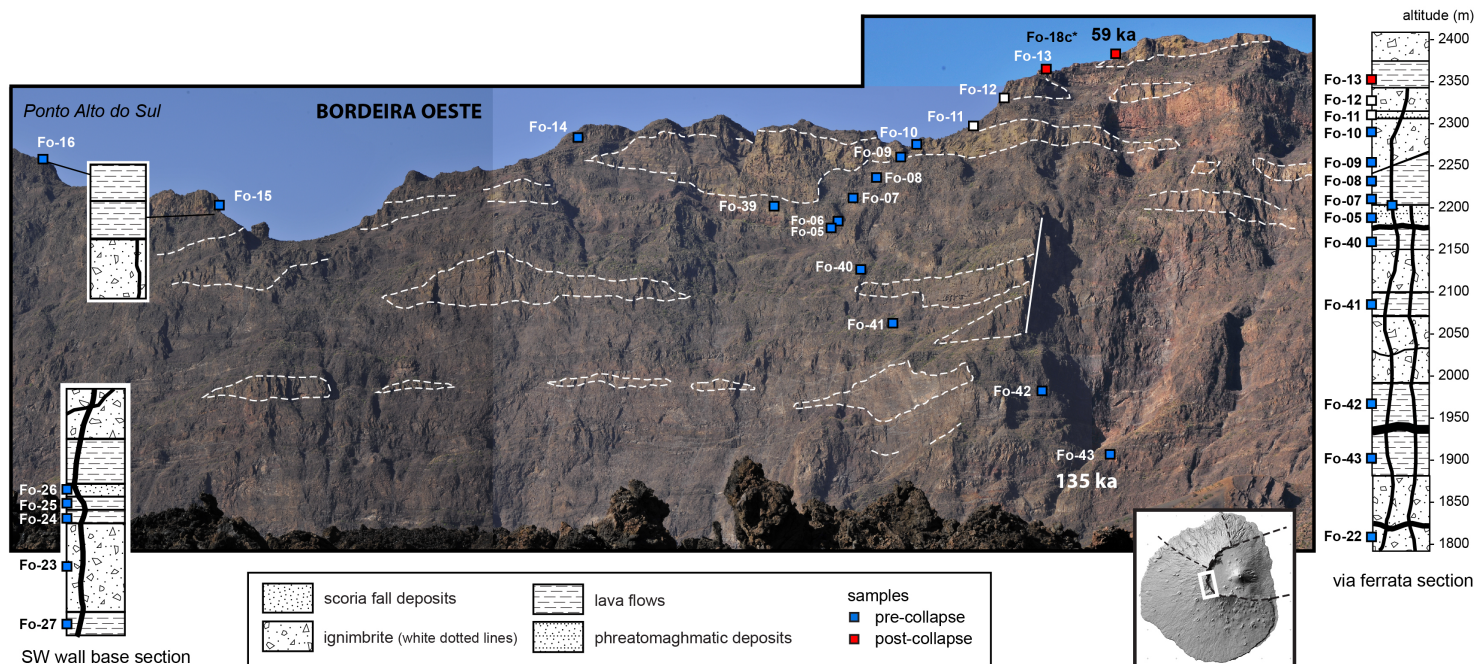

Supplement: Supplementary file 2 — Supplementary Figure S2. [file 41598_2021_96897_MOESM2_ESM.pdf]

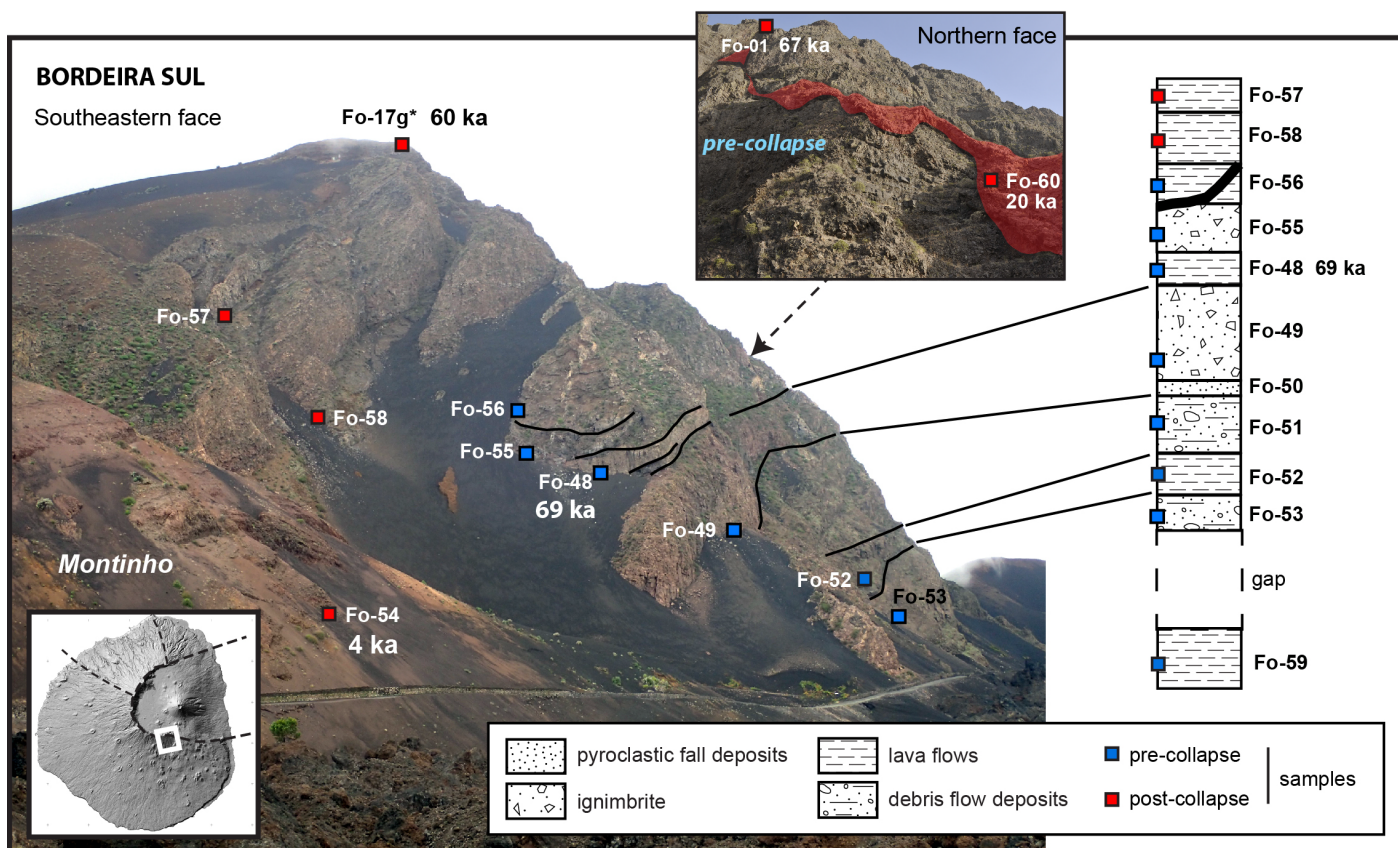

Supplement: Supplementary file 3 — Supplementary Figure S3. [file 41598_2021_96897_MOESM3_ESM.pdf]

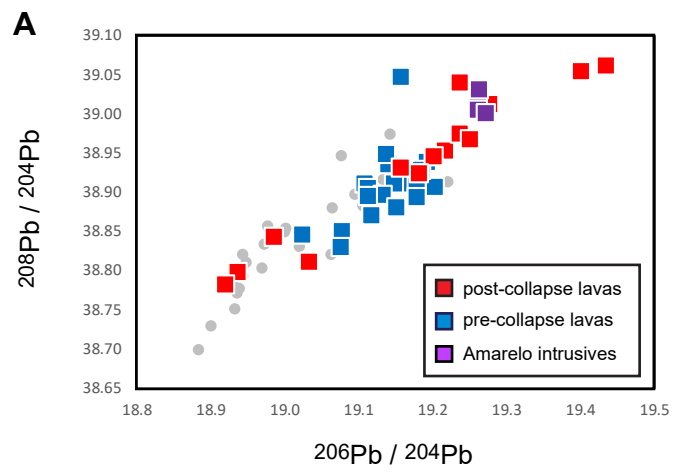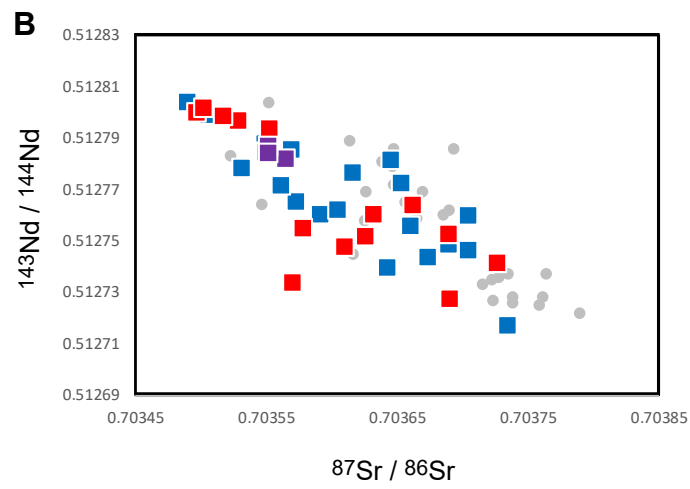

Supplement: Supplementary file 4 — Supplementary Figure S4. [file 41598_2021_96897_MOESM4_ESM.pdf]

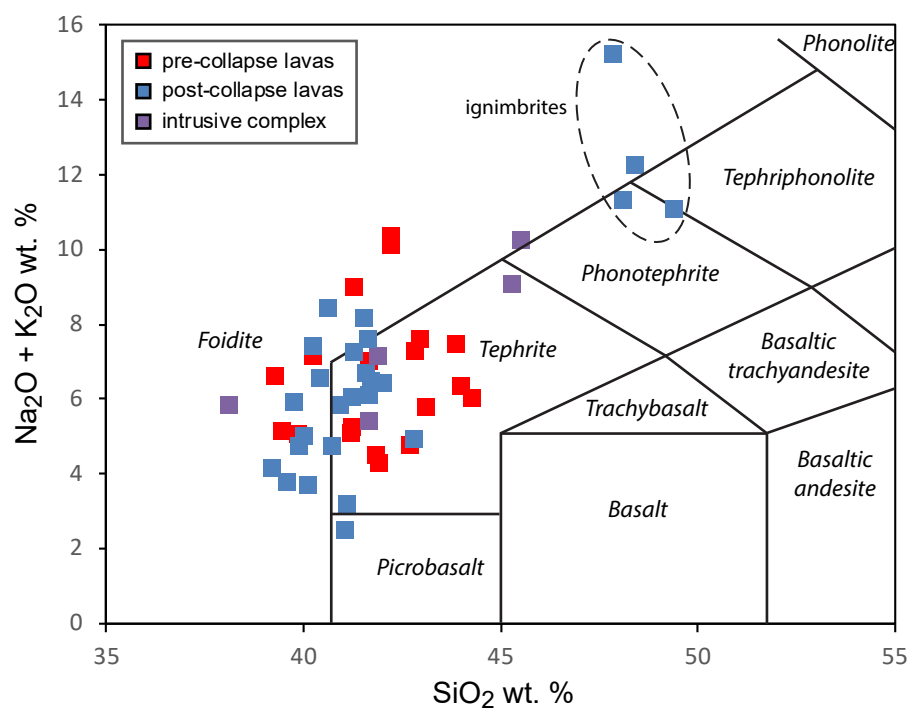

Supplement: Supplementary file 5 — Supplementary Figure S5. [file 41598_2021_96897_MOESM5_ESM.pdf]

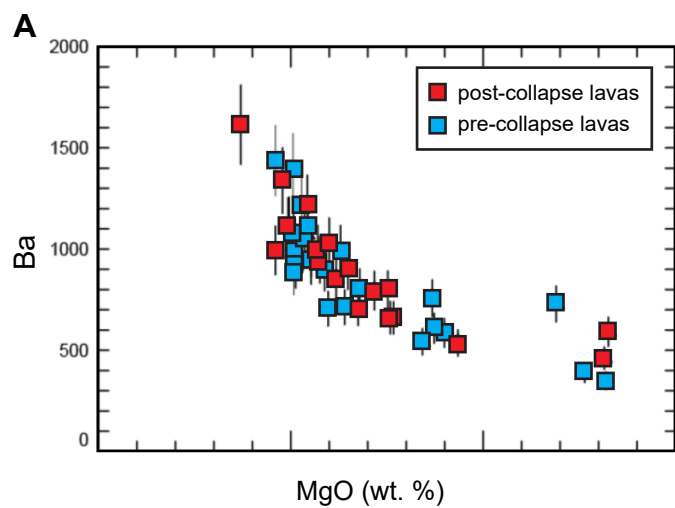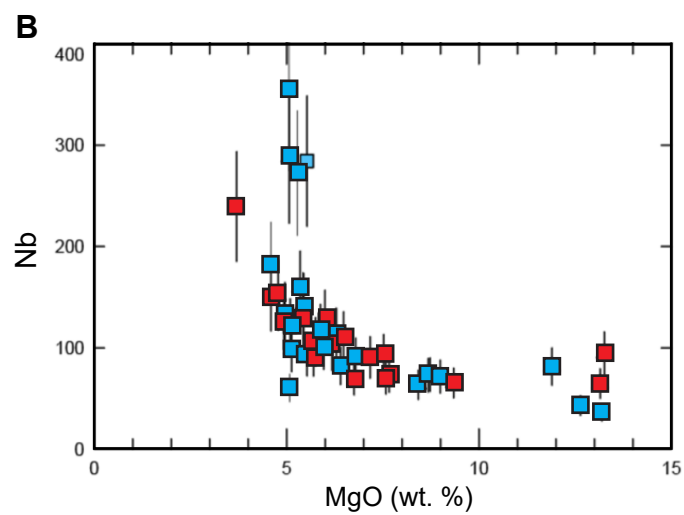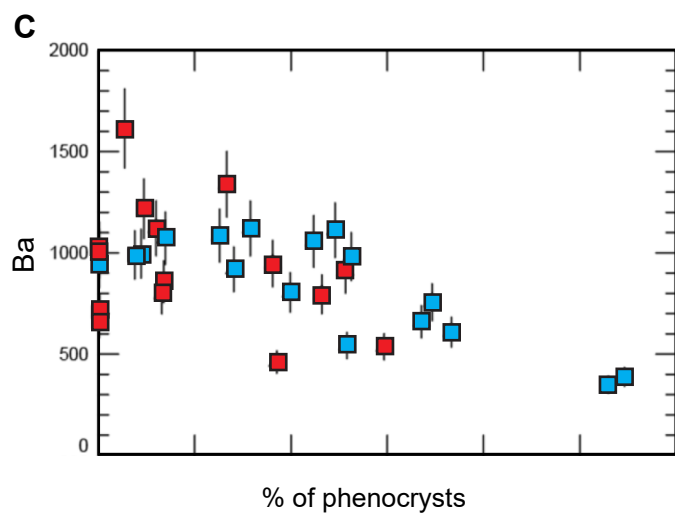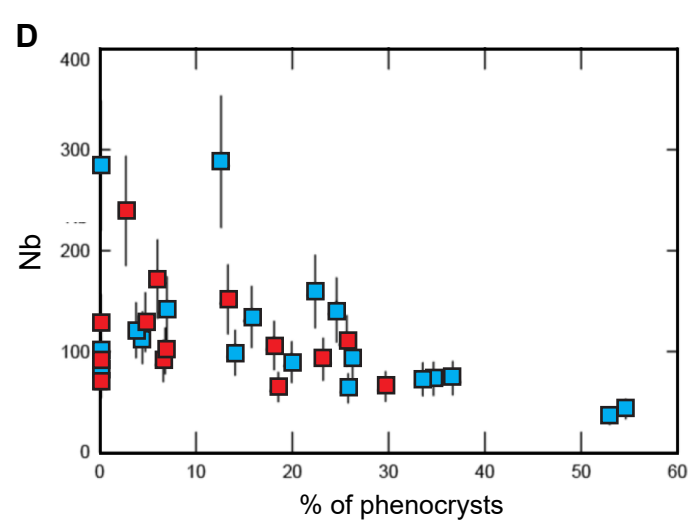

Supplement: Supplementary file 6 — Supplementary Figure S6. [file 41598_2021_96897_MOESM6_ESM.pdf]

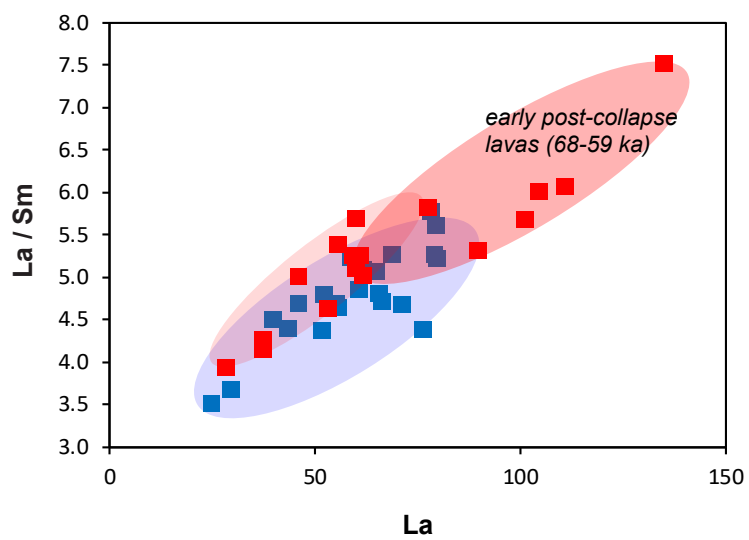

Supplement: Supplementary file 8 — Supplementary Figure S8. [file 41598_2021_96897_MOESM8_ESM.pdf]

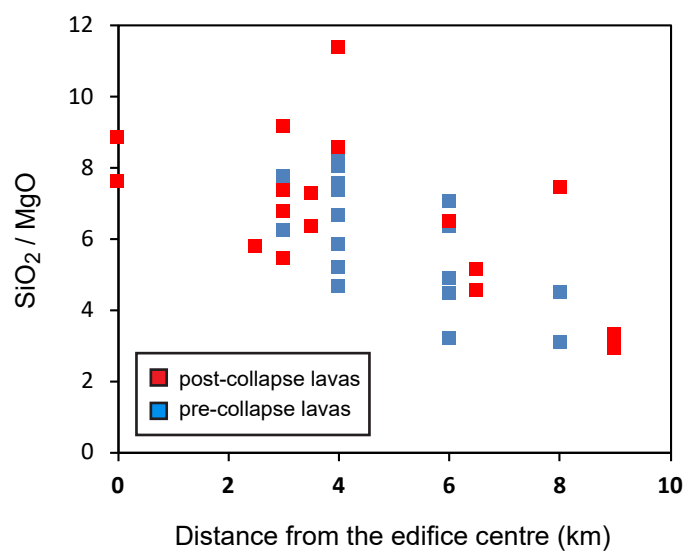

Supplement: Supplementary file 9 — Supplementary Figure S9. [file 41598_2021_96897_MOESM9_ESM.pdf]
