# Supplementary material for: Exploring the links between volcano flank collapse and the magmatic evolution of an ocean island volcano: Fogo, Cape Verde
Source: Sci Rep. 2021 Sep 1;11:17478. doi: 10.1038/s41598-021-96897-1 (PMC8410878; doi:10.1038/s41598-021-96897-1)
Supplement: Supplementary file 7 — Supplementary Figure S7. [file 41598_2021_96897_MOESM7_ESM.pdf]

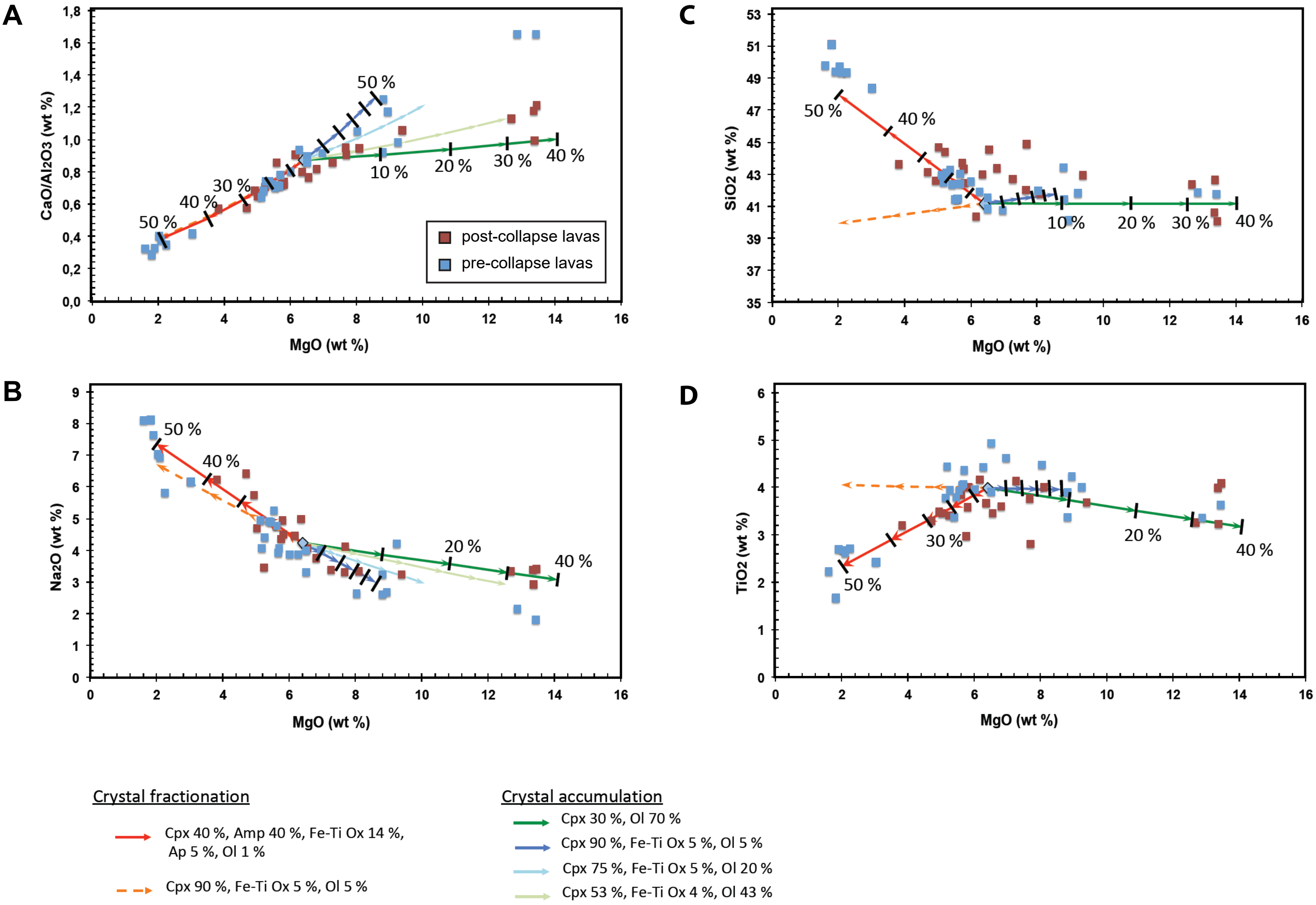
A
CaO/Al2O3 (wt %)
MgO (wt %)
post-collapse lavas
pre-collapse lavas
50 %
40 %
30 %
10 %
20 %
30 %
40 %
50 %
B
Na2O (wt %)
MgO (wt %)
50 %
40 %
20 %
40 %
C
SiO2 (wt %)
MgO (wt %)
50 %
40 %
10 %
20 %
30 %
40 %
D
TiO2 (wt %)
MgO (wt %)
30 %
50 %
20 %
40 %
Crystal fractionation
Cpx 40 %, Amp 40 %, Fe-Ti Ox 14 %, Ap 5 %, Ol 1 %
Cpx 90 %, Fe-Ti Ox 5 %, Ol 5 %
Crystal accumulation
Cpx 30 %, Ol 70 %
Cpx 90 %, Fe-Ti Ox 5 %, Ol 5 %
Cpx 75 %, Fe-Ti Ox 5 %, Ol 20 %
Cpx 53 %, Fe-Ti Ox 4 %, Ol 43 %
