# Supplementary material for: Exploring the links between volcano flank collapse and the magmatic evolution of an ocean island volcano: Fogo, Cape Verde
Source: Sci Rep. 2021 Sep 1;11:17478. doi: 10.1038/s41598-021-96897-1 (PMC8410878; doi:10.1038/s41598-021-96897-1)
Supplement: Supplementary file 10 — Supplementary Information. [file 41598_2021_96897_MOESM10_ESM.docx]

**Supplementary figure captions**

Fig. S1 – Sampling on the northern caldera wall. Lavas of the upper shield (160-70 ka) fill a paleo-collapse scar (the northwestern collapse) and they are cut by the Bordeira – Monte Amarelo collapse (~68 ka). Three types of post-collapse lavas are distinguished: (1) lavas on top of the Bordeira, which are sometimes eroded by post-collapse retrogressive erosion of the wall; (2) lavas overlapping the pre-collapse sequence of the Bordeira wall; (3) and lavas filling the caldera floor, including historical lavas.

Fig. S2 – Sampling on the western caldera wall. Most of the samples were collected along the via ferrata, with additional samples at the base and on top of the caldera wall. Note the recurrence of explosive products (ignimbrites) all along the pre-collapse sequence. *sample Fo-18c dated 59 ka is from Marques et al. (2019).

Fig. S3 – Sampling on the southern caldera wall allows the Monte Amarelo collapse to be dated accurately, with the younger pre-collapse lava at 69 ka and the older post-collapse lava at 67 ka. *sample Fo-17g dated 60 ka is from Marques et al. (2019).

Fig. S4 – ^206^Pb/^204^Pb *vs.* ^208^Pb/^204^Pb, and ^87^Sr/^86^Sr *vs.* ^143^Nd/^144^Nd plots, with pre-collapse samples in blue, post-collapse samples in red, intrusive samples in purple, and literature data in grey (Gerlach et al., 1988; Escrig et al., 2005; Mata et al., 2017).

Fig. S5 – TAS diagram of Fogo lavas. Whole rock major element composition of lava flows was analysed by ICP-AES, and glass composition of ignimbrites was analysed at the electron microprobe.

Fig. S6 – Trace elements abundance (in ppm) *vs.* MgO (wt. %) and proportion of phenocrysts (pyroxenes and olivines).

Fig. S7 – Mass balance estimates of crystal fractionation or accumulation as shown by MgO contents (wt. %) compared to (A) CaO/Al_2_O_3_, (B) Na_2_O (wt. %), (C) SiO_2_ (wt %), and (D) TiO_2_ (wt %). Mass balance calculations are based on average compositions of pyroxene, amphibole (kaersutite), olivine, apatite and Fe-Ti oxides. The reference sample is an aphyric lava (Fo-37 ~160 ka: blue diamond on the diagram) representing the least differentiated liquid. The removal of a mixture rich in clinopyroxene and amphibole (+ Fe-Ti oxides, apatite and olivine) most likely accounts for the compositional changes by fractional crystallization. The most porphyritic compositions are explained by the addition of a mixture of clinopyroxene and olivine (+Fe-Ti oxides) in variable proportions. Note that low-Mg samples (< 3% MgO) include ignimbrite samples analysed at the microbeam.

Fig. S8 - La/Sm *vs.* La plots of Fogo lavas with pre-collapse samples in blue and post-collapse samples in red. Note that early post-collapse lavas are characterized by higher La/Sm ratios and La contents. The similarity in the Sr-Nd-Pb isotopic ratios (Fig. 3) between the early post collapse lavas (68-59 ka) and the late pre-collapse ones (80-68 ka) shows that the enrichment in incompatible elements such as La (and concomitant La/Sm increase) is unrelated to the source heterogeneity, but rather suggests a low degree of partial melting.

Fig. S9 – SiO2 / MgO ratio of lavas compared to their distance from the paleo-centre of the Monte Amarelo edifice (reconstructed from dyke orientation: Marques et al., 2019).

**Supplementary table captions**

Table S1 – Excel spreadsheet with sampling, major elements, trace elements, and Sr-Nd-Pb isotope data.

Table S2 – K-Ar ages of samples from FOGO. Age calculations are based on the decay and abundance constants from Steiger and Jäger (1977). (*) Given the difficulties to achieve the gas clean-up, sample FO-28 was analysed only one time.

Table S3 – Summary of ^40^Ar/^39^Ar data from incremental heating experiments. Ages calculated relative to 1.184 Ma Alder Creek Rhyolite sanidine standard. F: Spreading factor of Jourdan et al. (2009).

**References cited in supplementary figure and table captions**

Escrig, S., Doucelance, R., Moreira, M. & Allègre, C. J. Os isotope systematics in Fogo Island: Evidence for lower continental crust fragments under the Cape Verde Southern Islands. *Chemical Geology* **219**, 93 113 (2005).

Gerlach, D. C., Cliff, R. A., Davies, G. R., Norry, M. & Hodgson, N. Magma sources of the Cape Verdes archipelago: Isotopic and trace element constraints. *Geochimica et Cosmochimica Acta* **52**, 2979 2992 (1988).

Jourdan, F., Renne, P.R., Reimold, W.U. An appraisal of the ages of terrestrial impact structures. *Earth and Planetary Science Letters* **286**, 1-13 (2009).

Mata, J. *et al.* The 2014–15 eruption and the short-term geochemical evolution of the Fogo volcano (Cape Verde): Evidence for small-scale mantle heterogeneity. *Lithos* **288–289**, 91–107 (2017).

Marques, F. O., Hildenbrand, A., Victória, S. S., Cunha, C. & Dias, P. Caldera or flank collapse in the Fogo volcano? What age? Consequences for risk assessment in volcanic islands. *Journal of Volcanology and Geothermal Research* **388**, 106686 (2019).

Pourmand, A., Dauphas, N., Ireland, T.J. A novel extraction chromatography and MC-ICP-MS technique for rapid analysis of REE, Sc and Y: Revising CI-chondrite and Post-Archean Australian Shale (PAAS) abundances. *Chemical Geology* **291**, 38–54 (2012).

Steiger, R.H., Jäger, E. Subcommission on geochronology: convention on the use of decay constants in geo- and cosmochronology. *Earth and Planetary Science Letters* **5**, 320-324 (1977).
