# Supplementary material for: Exploring the links between volcano flank collapse and the magmatic evolution of an ocean island volcano: Fogo, Cape Verde
Source: Sci Rep. 2021 Sep 1;11:17478. doi: 10.1038/s41598-021-96897-1 (PMC8410878; doi:10.1038/s41598-021-96897-1)
Supplement: Supplementary file 12 — Supplementary Table S2. [file 41598_2021_96897_MOESM12_ESM.doc]

Table S2- K-Ar ages of samples from FOGO. Age calculations are based on the decay and abundance constants from Steiger and Jäger (1977). (*) Given the difficulties to achieve the gas clean-up, sample FO-28 was analysed only one time.

| Sample IDExperience n° | Weight molten (g) | K* (wt.%) | ^40^Ar* (%) | ^40^Ar^*^ (10^-13^ mol/g) | ^40^Ar^*^  (weighted mean ±1σ) | **Age ± 2σ (ka)** |
| --- | --- | --- | --- | --- | --- | --- |
|  |  |  |  |  |  |  |
| *PRE-COLLAPSE* |  |  |  |  |  |  |
|  |  |  |  |  |  |  |
| **FO-36**  *0231* | 1.00814 | 1.717 ± 0.017 | 5.014 | 4.811 |  |  |
| *0233* | 1.02539 | “…………..” | 5.520 | 4.734 | 4.764 ± 0.073 | **160 ± 4** |
| **FO-43**  *0230* | 0.98774 | 1.838 ± 0.018 | 8.283 | 4.152 |  |  |
| *0235* | 1.51286 | “…………..” | 7.286 | 4.482 | 4.317 ± 0.067 | **135 ± 4** |
| **FO-31**  *0557* | 0.98934 | 1.395 ± 0.014 | 4.817 | 2.987 |  |  |
| *0558* | 1.00042 | “…………..” | 5.318 | 2.981 |  |  |
| *0560A* | 0.98177 | “…………..” | 4.890 | 2.870 | 2.947 ± 0.055 | **122 ± 4** |
| **FO-48**  *0172* | 0.98855 | 3.248 ± 0.032 | 3.495 | 3.889 |  |  |
| *0189* | 2.16991 | “…………..” | 3.306 | 3.840 | 3.869 ± 0.055 | **69 ± 2** |
|  |  |  |  |  |  |  |
| *POST-COLLAPSE* |  |  |  |  |  |  |
|  |  |  |  |  |  |  |
| **FO-28^(*)^** |  | | | | | |
| *0189* | 1.00320 | 2.075 ± 0.021 | 2.657 | - | 2.135 ± 0.177 | **59 ± 10** |
| **FO-73**  *0242* | 1.00319 | 1.479 ± 0.015 | 0.525 | 0.892 |  |  |
| *0253* | 0.99842 | “…………..” | 0.454 | 0.897 | 0.894 ± 0.074 | **35 ± 4** |
| **FO-44**  *0219* | 0.98205 | 2.218 ± 0.022 | 0.638 | 0.799 |  |  |
| *0227* | 2.00768 | “…………..” | 0.809 | 0.954 |  |  |
| *0243* | 2.00914 | “…………..” | 0.646 | 0.724 | 0.824 ± 0.041 | **21 ± 2** |
| **FO-60**  *0229* | 2.03916 | 1.397 ± 0.014 | 0.641 | 0.653 |  |  |
| *0244* | 2.01890 | “…………..” | 0.570 | 0.512 | 0.573 ± 0.032 | **20 ± 2** |
| **FO-54**  *0234* | 2.01468 | 1.743 ± 0.017 | 0.064 | 0.042 |  |  |
| *0241* | 2.49553 | “…………..” | 0.228 | 0.142 | 0.119 ± 0.036 | **4 ± 2** |
| **FO-70**  *0245* | 1.03422 | 2.202 ± 0.022 | -0.311 | >0 |  |  |
| *0254* | 2.26939 | “…………..” | -0.008 | >0 | - | - |
