# Supplementary material for: Exploring the links between volcano flank collapse and the magmatic evolution of an ocean island volcano: Fogo, Cape Verde
Source: Sci Rep. 2021 Sep 1;11:17478. doi: 10.1038/s41598-021-96897-1 (PMC8410878; doi:10.1038/s41598-021-96897-1)
Supplement: Supplementary file 13 — Supplementary Table S3. [file 41598_2021_96897_MOESM13_ESM.docx]

Table S3 - Summary of ^40^Ar/^39^Ar data from incremental heating experiments. Ages calculated relative to 1.184 Ma Alder Creek Rhyolite sanidine standard. F: Spreading factor of Jourdan et al. (2009).

|  |  |  |  |  |  | **Age Spectrum** | |  |  | **Isochron analysis** | |  |
| --- | --- | --- | --- | --- | --- | --- | --- | --- | --- | --- | --- | --- |
| **Sample ID** | wt. | K/Ca | Total Fusion | Increments | ^39^Ar | Age |  |  |  | ^40^Ar/ ^36^Ar | Age | F |
| Experiment no. | (mg) | (total) | Age (ka) ± 2δ | used (^o^C) | (%) | (ka) ± 2δ | MSWD | N | MSWD | intercept (ka) ± 2δ | (ka) ± 2δ |  |
|  |  |  |  |  |  |  |  |  |  |  |  |  |
| **FO-01** |  |  |  |  |  |  |  |  |  |  |  |  |
| FG-1616 to FG-1622 | 116 | 2.46 | 60.7 ± 10.4 | 599-946 | 92.9 | 68.1 ± 9.4 | 0.06 | 6 of 7 | 0.08 | 298.81 ± 4.9 | 66.5 ± 33.9 | 2.5 |
| FG-1623 to FG-1629 | 117 | 2.43 | 57.3 ± 18.4 | 599-947 | 92.7 | 62.9 ± 17.5 | 0.05 | 6 of 7 | 0.06 | 298.31 ± 7.7 | 64.7 ± 57.8 | 2.5 |
|  |  |  |  |  |  |  |  |  |  |  |  |  |
| *weighted mean plateau and isochron ages from two experiments:* | | | | | | **66.9 ± 8.3** |  |  |  | 298.7 ± 4.1 | **66.0 ± 29.0** | |
|  | | | | | |  |  |  |  |  |  | |
